# A Rare Presentation of Possible Disseminated Histoplasmosis with Adrenal Insufficiency Leading to Adrenal Crisis in an Immunocompetent Adult: A Case Report

**DOI:** 10.1155/2020/8506746

**Published:** 2020-03-17

**Authors:** Wanasinghe Arachchilage Praneeth Prabash Jayathilake, Kulatunga Wijekoon Mudiyanselage Pramitha Prabhashini Kumarihamy, Dissanayake Mudiyanselage Priyantha Udaya Kumara Ralapanawa, Widana Arachchilage Thilak Ananda Jayalath

**Affiliations:** ^1^University Medical Unit, Teaching Hospital Peradeniya, Kandy, Sri Lanka; ^2^Department of Medicine, University of Peradeniya, Peradeniya, Sri Lanka

## Abstract

Histoplasmosis is caused by *Histoplasma capsulatum*, and commonly it causes an asymptomatic illness. Although *Histoplasma* is the commonest organism to infect adrenal glands, disseminated histoplasmosis in an immune-competent host leading to adrenal insufficiency is rare in current literature. Here, we report a case of possible disseminated histoplasmosis leading to adrenal crisis in a young Asian immunocompetent male. A 42-year-old Sri Lankan male with noninsulin-dependent diabetes mellitus presented with constitutional symptoms and feverishness for three weeks' duration. He was found to have hepatosplenomegaly with bilateral adrenal masses and pancytopenia. One week later, he developed severe vomiting and hemodynamic collapse and was found to have suppressed adrenal functions. Ultrasound-guided biopsy of adrenals showed fungal spores morphologically similar to *Histoplasma*. He was started on oral itraconazole and adrenal replacement therapy. He improved symptomatically with treatment and currently is on regular clinic follow-up with a plan to continue antifungal therapy for at least one year to prevent a relapse. Fungal infections, especially histoplasmosis, need to be considered in all immunocompetent patients with compatible history and bilateral adrenal masses. Adrenal insufficiency needs to be promptly diagnosed and treated to prevent Addisonian crisis in these patients.

## 1. Introduction

Histoplasmosis is caused by a thermally dimorphic fungus *Histoplasma capsulatum*, which is rarely encountered in Sri Lanka. Mainly, it causes an asymptomatic self-limiting pulmonary infection [1–4] but can cause various clinical manifestations ranging from pulmonary histoplasmosis to progressive disseminated disease [3, 5]. Symptomatic disease is more common among immunosuppressed individuals. Adrenal disease is more commonly encountered in disseminated disease, and *Histoplasma* is the commonest fungus to infect the adrenal glands. But symptomatic adrenal insufficiency is rare. Here, we report a case of an immunocompetent patient presenting with possible disseminated histoplasmosis with adrenal involvement leading to adrenal insufficiency and Addisonian crisis which is a rare finding in clinical practice. According to our knowledge and literature review, this is the first documented case report of adrenal insufficiency due to possible disseminated histoplasmosis in Sri Lanka.

## 2. Case Presentation

A 42-year-old Sri Lankan grocery shop owner with noninsulin-dependent diabetes mellitus on diet control for three years duration admitted to the medical ward of a tertiary care hospital in central Sri Lanka with generalized malaise and feverish feeling for three weeks' duration. His feverishness was mainly at night, and it was associated with evening sweating and severe anorexia. He has lost three kilograms of body weight during that period. No chronic cough, hemoptysis, or contact history of tuberculosis was noted in the history. He had nausea, but there was no vomiting or abdominal pain. His urinary and bowel habits were normal. There was no arthralgia, morning stiffness, photosensitive rashes, or any other symptoms suggestive of any autoimmune disease.

He was diagnosed to have type 2 diabetes mellitus three years ago and on diet control with good glycemic control. He did not have any micro- or macrovascular complications related to diabetes mellitus. There were no past hospital admissions or episodes of recurrent urinary, respiratory, or skin sepsis. He owns a small grocery shop and works in there. He did not have significant exposures to birds, animals, paddy, and wet soil. His travel history was unremarkable. He had five pack-years of cigarette smoking and consumes alcohol occasionally. He is married and has two children. He denied any high-risk behaviors including sexual promiscuity or intravenous drug abuse.

On examination, he was averagely built, afebrile, and mildly pale. There was no cervical, axillary, or inguinal lymphadenopathy. No skin rashes or mouth ulcers to be seen. His ic fundi were normal. His pulse rate was 72 beats per minute (bpm) and blood pressure was 110/70 mmHg without any postural drop. His cardiovascular and respiratory system examinations were unremarkable. He had mild hepatomegaly which was nontender, and there was no associated splenomegaly or other palpable masses in the abdomen clinically.

Full blood count showed pancytopenia with a white blood cell count of 3 *∗* 10^9^/liter (l), platelet count of 120 *∗* 10^6^/l, and hemoglobin of 10 grams per deciliter (g/dl). C-reactive protein (CRP) was 8 and erythrocyte sedimentation rate (ESR) was 60 mm in the first hour. His liver and renal profiles including serum electrolytes were normal. 2D echocardiogram and chest X-ray were unremarkable. His septic screening including blood and urine cultures were negative. Mantoux test was negative. Hemoglobin A1c (HbA1c) was 6.5%. Ultrasound scan (USS) of the abdomen showed mild hepatosplenomegaly with bilateral hypoechogenic masses in the suprarenal glands with normal kidneys and few enlarged para-aortic lymph nodes. Although blood picture was compatible with pancytopenia, the bone marrow biopsy only showed reactive marrow without any evidence of leukemia, lymphoma, or secondary deposits. Bone marrow biopsy was negative for both fungal studies and tuberculosis.

After admitting to the ward, the patient has felt well with the symptomatic treatment. Meanwhile, an urgent contrast-enhanced computer tomography (CECT) chest and abdomen was arranged suspecting an adrenal neoplasm, but it was delayed due to a technical issue with the computer tomography (CT) machine. Since the patient was stable with the symptomatic treatment, he went home with a plan to review with the CECT report and random serum cortisol levels.

**Figure 1:**
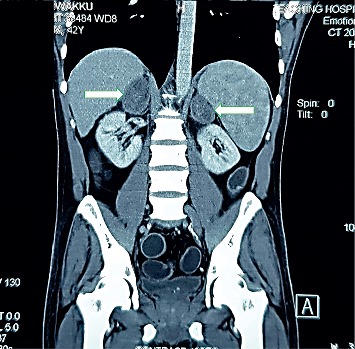
CECT abdomen showing bilateral adrenal masses (white arrows).

One week later, he was again admitted to the same medical unit with severe nausea, vomiting, diarrhea, abdominal pain, and postural dizziness for three days' duration. There was no associated fever or a history of food poisoning. On examination, he was dehydrated, and his pulse rate was 100 bpm, and blood pressure was 90/60 mmHg with 30 mmHg of postural drop in systolic blood pressure. The rest of the examination was normal. He was initially resuscitated with intravenous fluid boluses, but even after correcting his dehydration, his blood pressure remained low. Since he had bilateral adrenal masses, an Addisonian crisis was suspected, and he was started on intravenous hydrocortisone 200 mg bolus followed by 200 mg 8 hourly. With that, his blood pressure picked up and stabilized. His serum sodium value was 114 mmol/l, and serum potassium value was 5 mmol/l. Random blood sugar was 80 mg/dl, and corrected serum calcium level was marginally elevated. His random cortisol level was very low at 1.32 *μ*g/l. His full blood count showed improving pancytopenia. His renal functions and inflammatory markers were within normal range. An urgent CECT chest and abdomen revealed bilateral well-defined homogenously enhancing adrenal masses (left side-6.9 *∗* 3.7 *∗* 5 cm and right side-5.3 *∗* 3.4 *∗* 5.4 cm in size) with mild hepatosplenomegaly and patchy para-aortic lymphadenopathy (Figure 1). There were no other mass lesions in the abdomen and chest. Then, adrenocorticotrophic hormone (ACTH) stimulation test was performed after converting hydrocortisone to oral dexamethasone therapy, and it showed a persistently low serum cortisol value less than 1 *μ*g/l even after stimulating with synthetic ACTH preparation. So, Addison's disease was confirmed, and the patient was started on replacement steroid and mineralocorticoid therapy.

**Figure 2:**
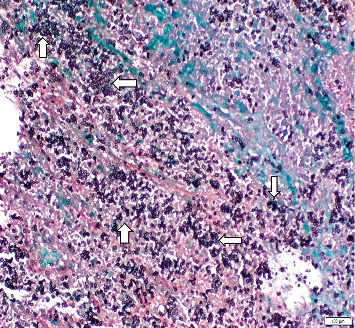
Gomori methenamine silver (GMS) stained adrenal biopsy specimen. Some of the fungal spore clusters are shown with white arrows.

USS-guided adrenal mass biopsy reveled a core tissue with necrosis at one end with infiltration of plasma cells and lymphocytes alone with histiocytes in adjacent tissues. With the Gomori–Grocott methenamine silver (GMS) staining, there were numerous fungal spores which were morphologically similar to *Histoplasma* in the necrotic and adjacent tissues (Figure 2). An upper gastrointestinal endoscopy was performed to see any evidence of disseminated histoplasmosis, and it was normal. His blood and tissue specimen was sent for fungal cultures. We did not do additional diagnostic investigations including serum and urine antigen assays, polymerase chain reaction assay, and specific cultures due to financial limitations and nonavailability of the facilities. His human immunodeficiency virus (HIV) 1 and 2 serology was negative.

The diagnosis of possible disseminated histoplasmosis with adrenal involvement leading to Addisonian crisis was made, and he was started on oral itraconazole 100 mg twice per day in addition to oral fludrocortisone 0.5 mg per day and oral hydrocortisone 10 mg at 6 am, 5 mg at noon, and 5 mg at 6 pm. With the treatment, his symptoms improved, and he started to gain weight. After six months of itraconazole therapy, the CECT abdomen and the adrenal biopsy were repeated, and it showed persistent bilateral adrenal masses and persistent *Histoplasma* infection histologically. So, the decision was taken to continue itraconazole therapy for at least one-year duration and thereafter to continue it further according to the radiological and histological response. The patient is regularly followed up in a medical clinic, and upto now he does not show any evidence of worsening of Addison's disease or any evidence of hepatotoxicity related to the itraconazole therapy. But he has developed generalized body pigmentation after about three months into the therapy although he was improving symptomatically (without any postural drop in systolic blood pressure, and his serum electrolytes were within normal range).

## 3. Discussion

Histoplasmosis is a granulomatous illness caused by the thermally dimorphic sacrophytic fungus *Histoplasma capsulatum*. It has a worldwide distribution. Although it is not endemic in Sri Lanka, *Histoplasma* is endemic in some parts of Southeast Asia, Australia, Central and Northern America, Eastern and Southern Europe, and Africa [1–3, 5]. Histoplasma grows as a mycelium at ambient temperatures especially in moist acidotic soils in river banks and caves and in bat and bird droppings [2–5]. When the microsporidia are inhaled, it changes its form and grows as a yeast within the alveolar macrophages. Then, it disseminates via reticuloendothelial system to other parts of the body [4].

Usually, innate immune response is mounted within two weeks and clears the infection from the body. Histoplasmosis is usually a self-limiting disease in immunocompetent individuals, but rarely it can cause progressive disseminated disease in pediatric, geriatric, and in immunocompromised populations [2, 4]. Normally, it presents as an asymptomatic self-limiting pulmonary infection or a mild flu-like illness [1–4]. Commonly, patients who develop symptoms are either immunosuppressed or have been exposed to a large dose of inoculum. Clinical spectrum of the disease can vary from primary pulmonary histoplasmosis, primary cutaneous histoplasmosis, and African histoplasmosis to progressive disseminated histoplasmosis [3, 5]. Progressive disseminated form causes a chronic illness in immunocompetent hosts while it appears as an acute disease in immunocompromised individuals [3]. Progressive disseminated histoplasmosis is more common among immunosuppressed, and it can involve any organ system including the reticuloendothelial system, lungs, bone marrow, kidneys, adrenal glands, gastrointestinal system, eyes, and brain [1–6]. This form has various clinical presentations but commonly it presents as fever, loss of weight, loss of appetite, nausea, and night sweats [1, 4]. Only few cases of disseminated histoplasmosis in immunocompetent patients are reported in the literature.

Although the adrenal gland is commonly involved in the disseminated disease, primary adrenal histoplasmosis has also been documented. Rarely, as in our patient, the adrenal gland can be the only organ with demonstrable disease. The commonest organ to be involved in immunocompetent host with disseminated disease is the adrenal gland [4], and *Histoplasma* is the commonest fungus to infect the adrenal glands [1]. Involvement of both adrenals is common in histoplasmosis as in our patient. Adrenal involvement can occur during the active dissemination phase or many years after the disease resolution. Primary adrenal histoplasmosis and adrenal involvement during disseminated disease is more common among males [1]. The reason for this is not yet understood. Adrenal involvement can vary from milder isolated foci of cortical parasitized macrophages to severe forms including granulomatous adrenal replacement and masses of calcified lesions that can mimic adrenal malignancy or tuberculosis [1]. Adrenal histoplasmosis leading to progressive adrenal destruction and calcification poses a unique danger to the patient due to risk of developing adrenal insufficiency. These patients will have the signs and symptoms of adrenal insufficiency such as severe nausea, vomiting, postural dizziness, hyperkalemia, hyponatremia, and eosinophilia in addition to the usual constitutional symptoms [1]. Similar to our patient, sometimes they can present with life threatening Addisonian crisis needing urgent resuscitation and steroid replacement therapy. As in this case, diagnosis of adrenal insufficiency is usually delayed because of the nonspecific symptoms and delayed presentation [4]. Frequently, patients with disseminated disease have hepatomegaly with or without splenomegaly and lymphadenopathy on clinical examination and radiographic examination [1, 3]. Also, they may develop pancytopenia due to the bone marrow involvement. Our patient also had hepatomegaly with mild splenomegaly and para-aortic lymphadenopathy indicating that he is suffering from possible disseminated disease. However, the bone marrow was negative for fungal stain in our case although he had pancytopenia.

Bilateral adrenal gland involvement can occur with many disease conditions, and the commonest causes are the metastatic diseases; lymphomas, sarcoidosis; adrenal hemorrhages and infections including tuberculosis, blastomycosis, coccidomycosis, and cryptococcosis [1, 4, 6]. As in our patient who presents with fever and weight loss for three weeks' duration, the tuberculosis is high up in the differential diagnosis because it is more common and endemic in Sri Lanka. So, in any patient with bilateral adrenal masses and compatible history; tuberculosis, lymphoma and metastatic diseases need to be excluded first. Since this patient's adrenal tissue culture is negative for tuberculosis, we excluded the possibility of disseminated tuberculosis. At the same time, his adrenal biopsy did not show any histological evidence of tuberculosis, lymphoma, or disseminated malignancy.

CECT or USS of the abdomen can be used to assess adrenal masses, but they will have varying degree of appearances depending on the amount of adrenal involvement by the organism [1, 3]. Bilateral symmetrical adrenal involvement with areas of hemorrhages and necrosis is typical for adrenal histoplasmosis [1]. Nevertheless, this appearance is also seen with adrenal metastasis and other fungal infections such as paracoccidomycosis [1]. The diagnosis of adrenal histoplasmosis can be confirmed cytopathologically by ultrasound-guided fine needle aspiration cytology [1, 6]. It is a very safe and effective way of diagnosing the condition. USS or CT-guided biopsy and periodic acid-Schiff (PAS) or GMS staining also can be used in some cases [1, 4, 7, 8]. Histoplasma serum and urine antigen assays, polymerase chain reaction assays, blood cultures, immunoprecipitation antibodies, and complement fixing antibodies also support the diagnosis [9]. Furthermore, the bone marrow and the biopsies from the skin lesions can be used in progressive disseminated illness to facilitate the diagnosis [1, 4]. But the fungal tissue culture is the gold standard confirmatory investigation [1]. We used GMS staining of the USS-guided adrenal tissues for the cytopathological diagnosis of histoplasmosis. We did not use further serological confirmatory tests due to cost and nonavailability of the facilities.

Advanced diabetes mellitus is an immunosuppressive status. We considered our patient immunocompetent since he had a good glycemic control (HbA1c of 6.5%) while on diet control without any micro- and macrovascular complications related to diabetes. Since his retroviral screening is negative and he did not give a history of recurrent infections, we did not do further investigations to assess his immune status.

Milder forms of histoplasmosis do not need treatment [1, 6]. Treatment for the disseminated disease, including adrenal histoplasmosis, is depending on the severity of the infection and the condition of the patient. Amphotericin is used initially in severe infections and later needs to be converted to maintenance long-term oral itraconazole therapy [1, 4, 8]. Our patient was started on oral itraconazole therapy which was available to us. The ideal duration for the maintenance therapy is debated, and in literature, we have found the uses of itraconazole for six months to two years with varying degree of results depending on the patient's condition. But long-term treatment is recommended to prevent relapses [1]. Generally, treatment is continued at least for one year. Since itraconazole is hepatotoxic, liver functions needs to be monitored regularly. Although adrenal function is not recoverable, there are some reports of patients who have gained adrenal functions after prolonged antifungal therapy [1]. In addition, fluconazole and ketoconazole can also be used as an alternative in moderate to severe disease [1, 10].

Up to now, our patient is followed up in a medical clinic for eight months, and he does not have any evidence of liver toxicity. We are planning to continue itraconazole for one-year duration and repeat the CECT abdomen and adrenal biopsy. Depending on the findings, we are planning to decide on further duration of antifungal therapy. Our patient is still having adrenal insufficiency indicating possible nonrecoverable adrenals. However, his adrenal function needs to be assessed again once antifungal treatment is over.

In summary, disseminated histoplasmosis can rarely occur in young healthy immunocompetent patients, and it needs to be considered in every patient with adrenal masses and a compatible history. Adrenal biopsy is useful in differentiating adrenal fungal infections from adrenal neoplasms and other common diseases which involves adrenal glands. At the same time, adrenal insufficiency has to be promptly ruled out in these patients to prevent the dreadful complication of Addisonian crisis and its related mortality and morbidity.

## Abbreviations

Bpm: Beats per minute
l: Liter
g/dl: Grams per deciliter
CRP: C-reactive protein
ESR: Erythrocyte sedimentation rate
HbA1c: Hemoglobin A1c
USS: Ultrasound scan
CECT: Contrast-enhanced computer tomography
CT: Computer tomography
ACTH: Adrenocorticotrophic hormone
GMS: Gomori–Grocott methenamine silver
HIV: Human immunodeficiency virus
PAS: Periodic acid-Schiff.

## References

[1] Gajendra S., Sharma R., Goel S. (2016). Adrenal histoplasmosis in immunocompetent patients presenting as adrenal insufficiency. *Turkish Journal of Pathology*.

[2] Turashvili G., Cunningham K. S. (2016). Bilateral adrenal histoplasmosis in a man with chronic alcoholism. *Journal of Microbiology, Immunology and Infection*.

[3] Vyas S., Das P., Radhika S. (2011). Adrenal histoplasmosis: an unusual cause of adrenomegaly. *Indian Journal of Nephrology*.

[4] Rog C. J., Rosen D. G., Gannon F. H. (2016). Bilateral adrenal histoplasmosis in an immunocompetent man from Texas. *Medical Mycology Case Reports*.

[5] Parvin R., Akm R. U. (2013). Bilateral adrenal histoplasmosis in an immunocompetent man. *Journal of General Practice*.

[6] Rana C., Krishnani N., Kumari N. (2011). Bilateral adrenal histoplasmosis in immunocompetent patients. *Diagnostic Cytopathology*.

[7] Larbcharoensub N., Boonsakan P., Aroonroch R. (2011). Adrenal histoplasmosis: a case series and review of the literature. *The Southeast Asian Journal of Tropical Medicine and Public Health*.

[8] Afsana F., Hossain K. N., Tareque A., Amin M. F., Pathan M. F. (2017). A case of adrenal histoplasmosis. *BIRDEM Medical Journal*.

[9] Falci D. R., Hoffmann E. R., Paskulin D. D., Pasqualotto A. C. (2017). Progressive disseminated histoplasmosis: a systematic review on the performance of non-culture-based diagnostic tests. *The Brazilian Journal of Infectious Diseases*.

[10] Zida A., Niamba P., Barro-Traoré F. (2015). Disseminated histoplasmosis caused by *Histoplasma capsulatum* var. *duboisii* in a non-HIV patient in Burkina Faso: case report. *Journal de Mycologie Médicale*.

